# A Concise History of the Principal Discoveries in Anatomy

**Published:** 1799-09

**Authors:** 


### *A Concise History of the principal Discoveries in Anatomy.*

[Continued from our last Number, pp. 60—69.]

§ 10. TO relate the most important discoveries in systematic order, it will be necessary to begin with *Osteology*. The most interesting discovery which was made in this branch of anatomy, related to the ear. MONDINI and his followers, fearing they should commit a crime by sawing through the *os temporis*, had consequently left this important organ of sensation entirely unexamined. ALEXANDER ACHILLINI, about the year 1480, discovered the two bones of the labyrinth, the malleus and the incus, and even then described their uses. BERENGAR had a more accurate notion of the use of these bones; he described the *septum auris*, and doubted whether it originated from the auditory nerve, or from the membranes of the brain. VESALIUS added to these the vestibulum of the labyrinth, which he called *forum metallicum*, and the manubrium of the malleus, of which STEPHANUS knew as little as of the auditory bones in general. The third bone of the ear, which we call *stapes*, was yet undiscovered. INGRASSIAS, EUSTACHIUS, COLUMBUS, and LEWIS COLLADO, a pupil of Vesalius, and Professor at Valencia, contend for the honour of this discovery. Ingrassias, however, seems to have the strongest claim to it; for he maintains that he ought to be considered as the discoverer, having demonstrated the existence of the *stapes* publicly at Naples, in the year 1546. This assertion gains additional probability, when we compare with it the testimony of the just FALLOPIUS, who obtained the first information of it in the year 1548, from a pupil of Ingrassias. Vesalius and COITER are likewise of opinion that Ingrassias was the real discoverer of it. It cannot, however, be denied that Eustachius himself may be allowed the merit of a second discovery of this bone; but Columbus and Collado are much too late with their pretended discovery, the account of the latter having been first published in the year 1555. Eustachius discovered the tube which is called after his name, and the spiral line: he has also given a very good description of the membranous zone of the cochlea. Fallopius was the first who denominated the membrane of the tympanum, and gave an excellent description of it.

He

He had a knowledge of the aqueous duct of the vestibulum, of the nervous duct of the pyramid, of the lamina spiralis, and of the scalæ of the cochlea, as well as of the foramen ovale. ARANZI also had examined these parts very minutely, for he describes the round condyle of the anterior crus of the incus. VOLCHER KOYTER gives a good description of the nervous duct in the pyramid of the temporal bone, one sulcus of which is pervaded by the optic nerve; he knew the round and oval fenestra, the labyrinth, the semicircular canals, and the long process of the incus. ALBERTI and PLATER have likewise given a very good description of these parts.

§. 11. GUIDO GUIDI was the first who caused the pyramidal sanguiferous ducts of the temporal bone *(sinus petrosi)* to be correctly drawn; but they had been before described by Fallopius. The *os basilare* was first examined more minutely by Berengar. He discovered the cavities in its basis *(sinus sphenoidei)*, which terminate in the upper duct of the nasal cavity, and are frequently connected with the ventricles of the brain, by a foramen in the *ephippium;* hence he accounts for the origin of colds in the head, by an accumulation of mucus in those ventricles. Vesalius indeed accurately described this bone, together with its wings (*alæ*) and points (*apices*); but he would not admit its immediate connection with the ventricles of the brain, which really does not exist in many instances. SYLVIUS insisted upon the aforementioned connection being always admitted. Fallopius at length discovered that such ventricles are often not found in infants, and Ingrassias described the *os basilare* so carefully, and particularly explained the different modifications which it forms with the other bones so clearly, that it is scarcely possible to give a better description. GALEN had maintained, that there is a fissure between the dental cells, or on the external and anterior part of the upper jaw-bone, which in animals separates the intermaxillare from the os palatinum, but is never found in human beings. Vesalius contradicted this assertion, but admitted that a fissure divided the *apophysis palatina ossis maxillaris superioris* towards the inside, and in that direction disappeared between the dens incisor and the angular tooth. SYLVIUS again confounded the two fissures with each other, and thereby perplexed himself. Ingrassias gave a tolerably good description of the lower ossa spongiosa, and according to Berengar, who was the first that made this discovery, the real perforation of the os cribriforme, which formerly served to account for colds in the head, was denied by several anatomists. Guidi caused a good drawing to be made of the articulated condyles of the inferior maxilla with their cartilaginous superficies, and ALBERTI was the first who described the *ossa wormiana*. The *os hyoides* was represented by Vesalius, and all those who copied his plates, much larger than it is in a natural state, because the pieces of bone which are found in the ligaments of the hyoides of aged people, were mistaken for necessary parts of the bone itself. This prejudice was exploded only, after Eustachius had more minutely investigated the subject.

§. 12. Ingrassias rejected the foramina in the first vertebra of the neck, which Galen, misled by the observations he had made in dissecting apes, had adopted. Ingrassias, on the contrary, proved that sulci were actually in the atlas of the vertebral artery, and that the superfices of the ligaments of the occiput formed cavities, in conjunction with the superfices of the ligaments of the atlas. Eustachius, however, defended Galen, by asserting that the foramina ought to be denominated sulci. The number of the pectoral bones occasioned another dispute, which was carried on with great acrimony between Vesalius and Sylvius.—Galen had adopted seven in the human skeleton, but Vesalius proved that there were only three, and that his opponent had again been misled by the skeleton of a monkey. Sylvius objected to the assertion, that men had been larger and taller in the time of Galen, and had seven pectoral bones, but that in this dwarfish century, three only could be found.—Fallopius and Eustachius, who thought that this evasion was very ridiculous, nevertheless asserted, that in the embryo the sternum was composed of seven cartilages, which formed only a single bone in adults, and that Galen, having probably divided it according to the number of the ribs, was excusable. Vesalius was the first who proved, in opposition to Galen, that the first rib was immoveably connected with the sternum: Columbus, however, maintained a contrary opinion, merely to contradict his instructor. Sylvius observed also in the middle breast-bone, the large and disproportionate foramen, which indeed is frequently found there. The number of the *ossa cruciata* also excited the attention of anatomists; as Galen had mentioned only three, though from five to six were actually found. Vesalius was the first who demonstrated this, and Eustachius expressed himself in this instance rather unfavourably with regard to Galen. Vesalius was likewise the first who, from accurate experience, refuted the supposition of an incorruptible bone in the heart. Ingrassias afterwards corroborated this refutation. The former asserted, that the bones of the hand are not totally destitute of medullary substance, as Galen had maintained; and Sylvius again endeavoured to refute his assertion, by the absurd argument that the bones in former times, had been firmer and harder, and consequently required no such substance. The anatomists, in the beginning of this century, differed in opinion concerning the number of bones in the tarsus. Achilinus reckoned in the year 1502 only five bones in the tarsus, tarſus, probably becauſe he had conſidered the oſſa cuneiformia as one; but in the following year he found all the ſeven. Veſalius rejected the large curvature which Galen aſcribed to the os humeri, and the os ilium; while Sylvius defended Galen, by aſſerting, that the bones had become more ſtraight by the modern mode of dreſs. He vindicated, in a ſimilar manner, Galen's neglect in deſcribing the cartilages of the extremities of the bones: "In former times," ſaid he, "the bones were more ſolid, and conſequently required no cartilages." Stephanus knew, and deſcribed HAVER's *Synovia* in the joints of the bones.

§. 13. With reſpect to *Myology*, anatomiſts began to examine the ſtructure and powers of the muſcular ſyſtem. Galen had maintained, that a muſcle was compoſed of tendinous and nervous fibres. Veſalius, on the contrary, demonſtrated that there was no proportion between the nerves and muſcles; that frequently large nerves with numerous ramifications pervade the ſmaller muſcles, while only a few nerves paſs through the ſtrongeſt muſcles of the human body, for inſtance, the heart; that tendons differ entirely from muſcles, and rather approximate to the nature of ligaments. The muſcular fibre has, according to his opinion, a ſelf-ſubſiſtent power, capable of primary motion; nor does it loſe its capability of action, if it be wounded in a longitudinal direction. Fallopius explained this opinion more perſpicuouſly, and proved particularly, that motion, in every inſtance, is exerted only where muſcular fibres pre-exiſt; that it depends not always excluſively on the direction of thoſe fibres; and that conſequently it cannot be maintained with the ancients, that the oblique fibres effected a retentive, while the tranſverſe cauſed the protruding motion. Columbus traced the nerves from their ramifications to the muſcular fibre, and ſhewed that the latter apparently often originates from the former. The muſcular membrane *(panniculus carnoſus)*, which Galen had endeavoured to find in the whole extent of the cutis, was aſcribed by Veſalius to particular animals only. Stephanus alſo denied its exiſtence in the human body, and Koyter demonſtrated that the porcupine, by means of this membrane, was enabled to convolve itſelf. The majority of the anatomiſts of this century agreed, that a peculiar membrane covered the muſcles, and ſeparated them from each other; and we are indebted to STENONIS for the refutation of this error.

Many of the ſeparate muſcles were at this period diſcovered and properly denominated; others were more accurately deſcribed, and the incorrect anatomical accounts of the ancients, as well as the difference between the human and animal muſcles, were pointed out. Stephanus, like his predeceſſors ceffors, miftook the frontal and occipital mufcle for the periofteum, which was furrounded only with much cellular texture and adipous membrane. Fallopius, however, defcribed it firft very minutely and correctly. The doctrine of the ophthalmic mufcles, and of their ufe, was ftill blended with many errors. Berengar enumerated fix pair, and a folitary one; the the latter, in animals, reftrains the eye, and winds itfelf clofely round the optic nerve: this mufcle he farther fuppofed to exift in man.—Even Vefalius believed in the exiftence of this mufcle, and befides confidered the fphincter of the eye-lids as two different mufcles. Fallopius refuted both thefe erroneous notions, and proved that the mufcle in queftion was found only in herbivorous animals; and that the fphincter of the eye-lids was only a fingle one. Vefalius, however, obftinately contended for the exiftence of this internal mufcle, and imputed its obfcurity, in fome human bodies, to leannefs. Columbus refuted him likewife. Aranzi difcovered the elevator mufcle of the upper eye-lid in the year 1548, while he was a pupil of Maggi; but Fallopius probably knew nothing of this mufcle at that time, as he claimed the merit of having difcovered it in the year 1553. Aranzi is wrong in afferting, that the mufculi recti of the aged arife from the os bafilare. Koyter difcovered the corrugator fupercilii, or contractor of the eye-brows.

§. 14. It was even at this time generally underftood, that the mufcles of the ear perform voluntary motion: one of the retractors of this organ was alfo difcovered; it was reprefented by Euftachius, and defcribed by Columbus. The mufcles of the internal auditory organs alfo became the objects of anatomical inveftigation. Euftachius very accurately defcribed the dilatator and depreffor of the membrana tympani, and the mufcle of the ftapes; and Koyter's defcription is alfo agreeable to nature. Aranzi knew the dilatator of the membrana tympani, but was ignorant whether it was an artery or a nerve: and VAROLI denied for a long time the exiftence of thefe mufcles, miftaking them for nerves lacerated in fawing through the temporal bone. He at length became fenfible of his error, at leaft with regard to the mufcles of the ftapes, and even maintained that they could be fpontaneoufly moved. Vefalius defcanted on internal mufcles of the nofe, which were to ferve the purpofe of contracting it; but Columbus objected that he had found thefe mufcles only in animals, and defcribed in their place the external contractors of the foramen nafale. POSTHIUS, on the contrary, affures us that the difcovery of Vefalius really could be corroborated by inftances in very mufcular fubjects. Vefalius has an equally high opinion of his difcovery of a concealed mufcle of the cavum oris, which is the pterygoideus internus.— Fallopius added the pterygoideus externus, and the circumflexus palati.

Vefalius

Vesalius also pretended to have discovered between the os hyoides and the epiglottis, muscles (*hyo-epiglottideos*), which however were considered by Fallopius and Columbus as non-entities. Fallopius, besides, admitted only four muscles of the tongue, the stylo-glossus, the genio-glossus, the hyo-glossus, and the lingualis; though Vesalius had mentioned a greater number. Fallopius has, however, committed a great mistake, in asserting that the stylo-pharingeus presses against the tongue. Eustachius for the first time, mentions the stylo-hyoideus, and Berengar the thyreo-epiglotticus. The omo-hyoideus which the ancient anatomists supposed to arise from the caracoid procefs of the scapula, was traced from the superior margin of this bone by Columbus, who refuted Galen's assertion, that this muscle served to put the shoulder in motion. Eustachius caused correct drawings to be made of the muscles of the head, the neck, and particularly those of the cervix. Vesalius erroneously derives the biceps muscle of the upper jaw from the processus styloideus, whereas it arises from the processus mammillaris. Fallopius describes the descending muscle of the neck, the discovery of which has been ascribed by some to DIEMERBROEK, and attributes to the subclavian muscle the power of drawing the first rib upwards. He denies, on the contrary, though perhaps unjustly, that the large serrated muscle contributes any thing towards respiration. Fallopius mentions three muscles, instead of the scalenus of which Galen speaks, and we now know of four or five. He describes the internal muscle of the breast (*sterno-costalis*) as consisting always of four parts, though this muscle is subject to the most diversified changes.

§. 15. The notions, which Vesalius had of the intercostal muscles and their uses, were yet extremely obscure: he knew, however, that the functions of the external cannot be opposed to those of the internal intercostal muscles, as described by Galen, namely, that the external intercostal muscles contract the cavity of the thorax, while the internal ones expand it. Vesalius justly remarks, that both merely draw the ribs together, alternately. Guidi, on the contrary, believed that the external intercostal muscles only give way to the exertion of the internal ones, without acting themselves; and even Aranzi was of opinion, that the intercostal muscles served only as a septum, and exercised no particular action, FABRICIUS coincides with Galen, but thinks that his real opinion is contained only in those passages in which he says, that the external intercostal muscles expand, and the internal ones contract the cavity of thorax. The other passages of Galen, in which the contrary is affirmed, Fabricius pronounces spurious. He thinks that the cavity of the thorax must be expanded by the ribs being drawn upwards. Amongst the abdominal muscles, those which descend obliquely, obliquely, together with the pyramidal muſcles, were well deſcribed by Fallopius; he alſo was acquainted with POUPART's ligament *(ligamentum inguinale)*. Piccolhuomini was the firſt anatomiſt who denominated the white line *(linea alba muſculorum abdominalium)*. The doctrine of the ſuperior extremities *(artus ſuperiores)* has obtained much more perſpicuity by CANNANI's repreſentation and deſcription. In the ſecond figure, the flexor ſublimis digitorum is repreſented, and divides itſelf *here* into five tendinous parts: the third contains the ulnaris internus; the eighteenth, the lumbrical muſcles, and the flexor of the little finger: the nineteenth contains the muſcle of the palm *(palmaris brevis)*, which VALVERDE afterwards copied, and which Fallopius aſcribed to Cannani as an important diſcovery. This muſcle was believed at firſt to expand the palm of the hand: we know, however, that it ſerves to contract it. The ſhort flexor of the thumb, the ſeven muſcles between the bones of the metacarpus, and the abductor of the little finger, have been, if not diſcovered, at leaſt deſcribed firſt in a perſpicuous manner by Cannani. It is to be regretted, that he in general repreſents the muſcles unnaturally thick and tumefied! We find, indeed, the coraco-brachialis *(perforatus Caſſerii)* previouſly, though rudely, delineated in the work of Veſalius; but Aranzi was the firſt who gave a correct deſcription of it. The muſcles of the ſura, and the tranſverſe muſcle of the *planta pedis*, were diſcovered by Sylvius, and Fabricius deſcribed the latter correctly. The long extenſor of the toes was diſcovered by Columbus, the pyriform muſcle of the os ilium by Fallopius, and Veſalius inveſtigated the uſe of the muſcle of the poples more minutely, and maintained that it did not produce a perceptible flexion of the tibia. Fabricius obſerved the two-fold organization of this muſcle.

§. 16. The greateſt and moſt important diſcoveries were made in *angiology*, and the farther improvement of it occaſioned the foundation of a new ſyſtem, which changed the whole of the theory and practice of medicine: the veins had hitherto been conſidered as the principal veſſels; the real blood, and the function of nutrition had been excluſively aſcribed to them, and they had likewiſe been uniformly placed firſt in the compends of anatomy. Veſalius himſelf adopts this arrangement, as the arteries appeared to him to be only the conductors of the vital ſpirits from the heart throughout the body; he treats of them after the veins, and far leſs minutely than of the latter. Although he obſerved, on applying the ligature to arteries, a ſwelling between the parts compreſſed and the heart, yet this was cauſed, in his opinion, by ſtopping the efflux of the blood from that organ. But perceiving that this ſwelling under ſimilar circumſtances did not take place in the veins, he imputed it principally to the accumuaccumulation of the vital principle which is combined with the circulating blood in the arteries. It was generally believed, that the blood circulated to and from the vessels, according to the stimulus existing in different parts of the body, and particularly, that respiration propels the blood into the vessels by inspiring, and restores it to the heart by expiration. Galen's opinion, that the veins originated in the liver, had been exploded long before that period. Vesalius, especially, defended the hypothesis of ARISTOTLE, that the vena cava proceeded from the heart, and SUSIUS had publicly and zealously defended the same theory at Bologna, in the year 1543. Sylvius, however, continued to appeal constantly to the infallible Galen; and even Columbus, Eustachius, and Fallopius, earnestly maintained that the vena cava originated in the liver, and that in ascending it communicated with the heart by only one branch. It was supposed that the vena cava was united in the liver with the vena portæ, by forming considerable anastomoses, which, in this century, were generally conjectured to abound in the body. Varoli and DU LAURENS seriously endeavoured to prove the existence of these extensive anastomoses between the vena portæ and the vena cava. Berengar had formerly conjectured the existence of such large inosculations between the seminal arteries and their veins. Eustachius united, in a similar manner, the hypogastric veins with those of the bladder; and Fallopius did the same with the arteries of the mesentery and those of the rectum. It was generally believed, in imitation of Galen, that anastomoses subsisted between the veins of the breasts and those of the abdomen; with a view to account for the consensus between the uterus and the breasts. These inosculations are indeed very perceptible and easily demonstrated.

§. 17. More minute investigations into the nature of the valves of the larger vessels of the heart, and of the veins themselves, at that time occasioned deep reflections on the use of these membranes, and gradually opened the way to the theory of the circulation of the blood. We are indeed much indebted to Berengar in this respect. He described the semi-lunar valve of the ascending vena cava, and the valvulæ mitrales of the pulmonary vein. They appeared to him similar, because they did not fit completely, but shewed some laxity, were not possessed of equal firmness with the other valves, and always became contracted after the dilatation of the heart. He farther discovered the large tricuspidal valve between the oracle of the vena cava and the right ventricle of the heart, the use of which appeared to him to be the prevention of the blood in the heart, from its reflux into the cyst of the vena cava. He also described the arterial semilunar valves in the pulmonary artery and in the aorta, shewed the similarity of their structure, and supposed that, as they opened towards the heart, their function was to prevent the reflux of the blood. Sylvius had likewise observed the semilunar valve in the lower ascending vena cava, which erroneously has been denomidenominated after Euſtachius, who indeed has deſcribed it, and given an imperfect repreſentation, but certainly did not diſcover it. Veſalius has, by miſtake, been conſidered as the diſcoverer of the mitral valves in the pulmonary vein, though he deſcribes them better than Berengar; and he alſo ſhewed to Sylvius the valves in the aorta. Fallopius and Le Vaſſeur likewiſe knew the valves in the aſcending vena cava, in the cyſt of that veſſel, and the arterial veins; and they were of the ſame opinion with Berengar, reſpecting the functions of thoſe valves. Poſthius expreſſed himſelf ſtill more clearly with regard to the uſe of the valves in the large veins of the heart; and Aranzi deſcribed the cartilaginous edge of the valve in the pulmonary artery, and the little condyles of the mitral valves, which go by his name.

Valves were alſo diſcovered in ſeveral other veins. However, a ſeries of years elapſed before the moſt accurate reſult was deduced from theſe obſervations. It has already been mentioned, that Cannani, in the year 1547, diſcovered on the orifice of the ſolitary vein (*vena ſine pari*), a valve, which he ſuppoſed to be deſigned for preventing the ſuperabundant influx of blood from the vena cava. Sylvius had previouſly obſerved ſimilar valves in ſeveral other veins: but anatomiſts, nevertheleſs, treated Cannani's diſcovery with ridicule. Stephanus was, perhaps, the firſt who made this diſcovery, as he wrote prior to the year 1536, and hence deſerves the greateſt credit. Euſtachius clearly perceived the valves in the coronary veins of the heart, of which he cauſed drawings to be made. In the crural veins, Poſthius diſtinctly obſerved the valves in the year 1560, on the Anatomical Theatre of Montpellier; and a few years afterwards, Sal. Alberti made a ſimilar diſcovery in the renal as well as the crural and other veins. In the year 1574, Paul Sarpi and Fabricius obſerved thoſe valves in moſt of the veins of the human body; the latter furniſhed incomparable drawings of them, and explained their uſe, by ſtating that they were deſigned by Nature to prevent congeſtions, as well as too great dilatations of the veins. "In the arteries," ſaid Fabricius, "the valves were unneceſſary; as the influx and reflux of the blood here are not ſo much interrupted as in the veins. The valves are indiſpenſable to the veins of the limbs; becauſe, without them, irregularities might eaſily ariſe in thoſe parts, on account of their varied and almoſt inceſſant motion. The veins of the brain and the pelvis require no valves, as the blood naturally flows with greater velocity into theſe parts." From this paſſage it is obvious, that Fabricius did not conceive the principal uſe of the valves;—namely, that of promoting the return of the blood to the heart; a diſcovery of the firſt importance, which ſeems to have been reſerved for the ſuperior genius of the immortal Harvey.

[To be resumed in our next Number.]

